# Les déterminants du statut “perdu de vue” chez les patients pris en charge pour cancer au Maroc: situation avant le Plan Cancer

**DOI:** 10.11604/pamj.2014.18.83.2487

**Published:** 2014-05-25

**Authors:** Adil Najdi, Mohamed Berraho, Karima Bendahhou, Majdouline Obtel, Ahmed Zidouh, Hassan Errihani, Chakib Nejjari

**Affiliations:** 1 Laboratoire d'Epidémiologie, Recherche Clinique et Santé Communautaire; Fès – Maroc; 2 Equipe “Epidémiologie de la Prévention des Cancers” INSERM 897 ISPED; Bordeaux – France; 3 Direction Régionale de la santé, Casablanca, Maroc; 4 Direction de l'Epidémiologie et de lute contre les maladies, ministère de la santé. Maroc; 5 Association Lalla Salama de Lutte contre le cancer (ALSC); 6 Institut National d'Oncologie (INO) – Rabat

**Keywords:** Cancer, Perdu de vue, suivi, Maroc, Cancer, lost to follow-up, follow-up, Morocco

## Abstract

**Introduction:**

Le cancer au Maroc représente un problème majeur de santé publique, sa prise en charge doit être globale, active et complète pour tous les patients. L'objectif de ce travail était d'estimer la fréquence des perdus de vue « PDV » en oncologie au Maroc durant la première année de suivi et de déterminer les facteurs associés à ce problème.

**Méthodes:**

Par une étude rétrospective portant sur 2854 dossiers de malades hospitalisés dans les trois principaux centres d'oncologie au Maroc depuis janvier 2003 jusqu’à juin 2007 et concernant les cinq principales localisations de cancer au Maroc, nous avons cherché la date des dernières nouvelles des patients ayant un recul de 18 mois minimum afin de déterminer le statut de ces malades après un an de suivi.

**Résultats:**

La moyenne d’âge était de 52±14 ans, une proportion féminine de 63%, les sujets actifs constituaient 28%, les mariés 71%, les analphabètes 51%, 70% des patients habitaient en milieu urbain et seulement 11% des malades disposaient d'une couverture sociale. La localisation cancéreuse la plus fréquente était le poumon (23,8%) suivie du colon-rectum (23,5%) puis le col (21,9%), le sein (20,4%) et les lymphomes (10,4%). Le taux des «PDV» à un an de suivi était de 48%, ce statut était significativement lié au sexe, à l’âge, au NSE et au statut matrimonial. Sur le plan médical, le statut «PDV» était lié à la localisation du cancer, au stade de diagnostic et au type de traitement reçu.

**Conclusion:**

Notre étude a mis en évidence la grande ampleur du problème des PDV en cancérologie au Maroc ainsi que ces déterminants. Ces résultats incitent tous les acteurs dans le domaine de la cancérologie à collaborer ensemble pour prendre les mesures qui s'imposent pour y pallier

## Introduction

Plus de 22 millions de personnes dans le monde sont actuellement atteintes d'un cancer et environ 10 millions se voient diagnostiquer un cancer chaque année [1]. D'autre part, plus de 7 millions de personnes décèdent chaque année à cause de cette maladie. Dans des pays à moyen niveau économique comme le Maroc, on parle aujourd'hui de « transition épidémiologique ». En effet, on constate dans ces pays, avec l’élévation du niveau de vie, le changement des habitudes de vies (alimentation, activité physique…), les changements de l'activité économique et l'augmentation de l'espérance de vie, l’émergence de pathologies chroniques jusqu'ici très marginales notamment les pathologies endocriniennes, cardiovasculaires et néoplasiques [2]. De ce fait, les autorités de santé publique se trouvent dans la nécessité de prendre en considération une maladie comme le cancer jusqu'ici considéré comme une pathologie des pays riche, non prioritaire et de mesurer l'ampleur de son atteinte dans les populations [3].

Au Maroc, on estime que le cancer représente aujourd'hui un problème sanitaire majeur nécessitant une politique globale de prise en charge. En effet, selon le Registre des Cancers de la Grande Région de Casablanca (RCRC) 2004, l'estimation des nouveaux cas de cancer actuellement au Maroc est de 30 500/an [4]. Il apparaît actuellement au Maroc que, le cancer du sein avec une incidence standardisée de 35,04 pour 100 000 femmes/an, suivi du cancer du col de l'utérus avec une incidence standardisée de 13,46 pour 100 000 femmes/an constituent les localisations les plus fréquentes chez la femme. Chez les hommes, les cancers les plus fréquents sont le cancer du poumon suivi du cancer de la prostate avec une incidence standardisée de 25,5 et 9,6 pour 100 000 hommes/an respectivement. Pour les deux sexes, les cancers les plus fréquents sont les cancers colorectaux et les lymphomes tous les âges confondus [4].

Par ailleurs, en plus de l'accélération de jour en jour de son incidence, la maladie cancéreuse, est caractérisée par une issue défavorable en absence de traitement ce qui implique la nécessité d'un suivi médical non seulement long et coûteux mais aussi obligatoirement mené à terme, condition incontournable pour améliorer le pronostic.

D'autre part, et afin de pouvoir évaluer les efforts fournis dans cette lutte, nous avons besoin de connaître le devenir des malades, c'est-à-dire leur statut à des termes précis de leur évolution à savoir: vivant sans maladie, vivant et toujours malade ou décédé. Cependant le principal biais susceptible de compromettre cette évaluation est la présence d'un taux important de perdus de vue « PDV » c'est à dire de malades pour lesquels on ne dispose d'aucune nouvelle concernant leur statut vital ou vis-à-vis de leur maladie une fois que le diagnostic positif a été fait.

L'audit national préalable à un Plan national de lutte et de prévention des cancers réalisé au Maroc [1] par le Laboratoire d'Epidémiologie, Recherche clinique et Santé communautaire de Fès sur les stades de diagnostic de cancer a particulièrement porté son attention sur ce problème des PDV souvent évoqué par les différents praticiens exerçant dans le domaine du cancer. En effet ces praticiens estiment qu'un nombre non négligeable de malades cancéreux échappent au suivi.

L'objectif principal de cette étude est d'estimer la fréquence des PDV au cours de la première année de suivi dans les principaux centres de prise en charge du cancer au Maroc et de déterminer les facteurs liés au statut « PDV» à partir des données d'une étude rétrospective sur les stades de diagnostic et les résultats des traitements des cancers au Maroc.

## Méthodes

### Type d'enquête et population d’étude

Il s'agit d'une étude rétrospective sur les données des dossiers d'hospitalisation. Cette étude a été réalisée dans trois centres publics, l'Institut National d'Oncologie (INO) de Rabat, le Centre d'Oncologie de l'Hôpital Ibn Rochd de Casablanca et le service d'Hématologie de l'Hôpital 20 Août de Casablanca.

Nous avons fixé comme effectif d'inclure rétrospectivement dans cette étude 10% des cas de cancer hospitalisé dans les centres sélectionnée pour la réalisation de cette étude. Ainsi et sur l'estimation approximative de l'existence de 40000 dossiers, pour les cinq types de cancers les plus fréquents au Maroc, dans les trois centres sélectionnés pour la réalisation de cette étude pendant quatre années et demi (janvier 2003 à juin 2007); 4000 dossiers ont été tiré au sort. Et après exploitation de ces dossiers, seul 3928 dossiers contenant le stade de diagnostic ont été retenus. Les autres dossiers ne contenaient pas suffisamment de données exploitables.

La sélection des dossiers était basée sur un échantillonnage par choix raisonné en prenant en compte le centre d’étude et la localisation du cancer. En effet, pour chaque localisation, dans chaque centre et pour chaque année, 100 dossiers ont été tirés au sort à partir du registre d'hospitalisations.

Pour la présente étude, seuls les dossiers contenant l'information sur la date d'inclusion et la date de la dernière nouvelle (DDN) ont été retenus. Nous avons aussi dû éliminer les dossiers du premier semestre 2007 pour lesquels le recul ne dépassait pas 06 mois. Au total 2854 dossiers ont finalement pu être retenus.

### Définition des cas

Le sujet a été considéré comme PDV si le délai entre la date d'inclusion et la DDN était inférieur à un (01) an et pendant l'année qui a suivi la DDN on ne disposait d'aucune information sur le patient recueillie sur le dossier d'hospitalisation. Ont été exclus de l’étude les dossiers des enfants dont l’âge était < 16 ans (55 dossiers), les dossiers qui ne contenaient pas la date d'inclusion et/ou la date de dernière nouvelle.

### Recueil des données

Les informations ont été recueillies à partir des dossiers des malades pris en charge entre janvier 2003 et Décembre 2006. Elles comprennent: des données sociodémographiques (âge, sexe, niveau d’étude, profession, couverture sociale,….), les antécédents personnels et familiaux de cancer, les types de localisation du cancer, les classifications (TNM, autres), les traitements reçus (Chirurgie/Radiothérapie/ Chimiothérapie /Autres traitements), l'origine des patients (régions, provinces, milieu). De plus pour ce travail, les distances qui séparent les lieux d'origine aux lieux de diagnostic et du traitement ont été calculées pour chaque patient inclus dans l’étude. On a enfin recueilli le suivi et le devenir des patients après une année de suivi (Rémission, Guérison / Rechute / Décès / PDV).

Le recueil des données a été réalisé par un groupe d'enquêteurs formés à cet effet à partir des dossiers de patients classés dans les archives des centres concernés par l’étude, au moyen d'une fiche standardisée contenant les différentes informations à recueillir. Les enquêteurs (Médecins internes, médecins résidents et externes) ont été recrutés dans les services hospitaliers des centres d'enquête.

### Analyse des données

Les données recueillies ont été codées, saisies, puis validées pour être analysées. L'analyse a consisté en une description de l’échantillon selon la localisation du cancer, les caractéristiques sociodémographiques, les stades du diagnostic, les distances entre les lieux d'origine des patients et les lieux de diagnostic et de traitement, le type de traitement reçu, et le statut à un an de suivi et notamment le taux des PDV à un an. Les facteurs susceptibles d'influencer le statut PDV ont fait l'objet d'une analyse univariée permettant de tester les éventuelles associations, utilisant les tests chi2 et Student avec un risque d'erreur consenti a à 5%.

## Résultats

### Distribution des facteurs sociodémographiques et cliniques (Tableau 1, Tableau 2)

Sur les 2854 dossiers inclus dans l’étude, la distribution selon la localisation du cancer était comme suit: col (21,9%), sein (20,4%), poumon (23,8%), colon-rectum (23,5%) et lymphome (10,4%).

**Tableau 1:** Distribution des caractéristiques sociodémographiques de la population d’étude (N = 2854)

	Effectif	%
**Age (ans)**		
<40	595	21
40-50	1171	41
50-70	793	28
>70	276	10
**Sexe**		
Féminin	1806	63
Masculin	1042	37
**Statut professionnel**		
Actif	528	28
Inactif	325	17
Femme au foyer	1016	55
**Statut matrimonial**		
Célibataire	245	12
Marié	1503	71
Divorcé, veuf	365	17
**Origine**		
Urbain	1297	70
Rural	546	30
Couverture sociale		
mutualiste	183	11
Non mutualiste	1464	89
**Niveau socio-économique**		
bas	758	91
Moyen, haut	76	9
Niveau d’étude		
Analphabète	69	51
Non analphabète	66	49

**Tableau 2:** Caractéristiques médicales de la population d’étude (N = 2854)

	Effectif	%
**Antécédents personnels de cancer**		
oui	28	1
non	2772	99
**Antécédents familiaux de cancer**		
oui	130	5
non	2667	95
**Localisation de cancer**		
Poumon	626	22
Colon-rectum	581	20
col	680	24
sein	670	24
lymphome	296	10
**Stade diagnostic**		
Stade0-1	281	12
Stade2	627	27
Stade3	795	34
Stade4	631	27
**Type de traitement reçu**		
Chimiothérapie seule	461	19
Radiothérapie seule	336	14
Chirurgie seule	166	7
Chirurgie et chimiothérapie	343	14
Chirurgie et chimiothérapie et radiothérapie	619	25
Chirurgie et radiothérapie	160	6
Radiothérapie et chimiothérapie	394	16

La moyenne d’âge était de 52±13,9 ans, le sexe féminin représentait 63% de l’échantillon, 28% des patients étaient professionnellement actifs, 71% mariés, et seulement 11% étaient affiliés à une mutuelle pour la prise en charge des soins. La majorité des patients (70%) vivaient en milieu urbain et 51% des patients étaient analphabètes.

Des antécédents personnels de cancer ont été trouvés chez 1% des patients et des antécédents familiaux chez moins de 5% des cas. Le diagnostic a été fait aux stades 3 ou 4 chez 61% des patients.

A un an de suivi, 48% des patients étaient perdus de vue, 51% étaient vivants et 1% décédés tous en milieu hospitalier.

### Facteurs associés au statut PDV (Tableau 3)

La localisation cancéreuse la plus liée au statut PDV est la localisation pulmonaire avec un taux avoisinant 85% suivie de la localisation colorectale avec 57%. On note ensuite le col utérin avec 43%, le sein avec 27% puis les lymphomes avec 17% des PDV (p<10-3).

**Tableau 3:** Répartition des PDV selon les différents variables explicatives

	Taux des PDV(%)	p
**Age (ans)**		<10^-3^
<20	38	
20-40	46	
40-60	54	
>60	67	
**Sexe**		<10^-3^
Féminin	39	
Masculin	66	
**Statut professionnel**		<10^-3^
Actif	56	
Inactif	64	
Femme au foyer	42	
**Statut matrimonial**		<10^-3^
Célibataire	34	
Marié	50	
Divorcé, veuf	42	
**Origine**		0,30
Urbain	51	
Rural	53	
**Couverture sociale**		0,12
Mutualiste	42	
Non mutualiste	48	
**Niveau socio-économique**		<10^-3^
Bas	58	
Moyen, haut	38	
**Niveau d’étude**		0,81
Analphabète	22	
Non analphabète	20	
**Antécédents personnels de cancer**		0,80
Oui	49	
Non	46	
**Antécédents familiaux de cancer**		<10^-3^
Oui	34	
Non	49	
**Localisation de cancer**		<10^-3^
Poumon	85	
Colon-rectum	57	
Col	43	
Sein	27	
Lymphome	17	
**Stade diagnostic**		<10^-3^
Stade0-1	35	
Stade2	39	
Stade3	56	
Stade4	67	
**Type de traitement reçu**		<10^-3^
Chimiothérapie seule	52	
Radiothérapie seule	73	
Chirurgie seule	57	
Chirurgie et chimiothérapie	38	
Chirurgie et chimiothérapie et radiothérapie	17	
Chirurgie et radiothérapie	34	
Radiothérapie et chimiothérapie	40	

Le taux des PDV était particulièrement élevé pour le sexe masculin: 66% vs 39% pour le sexe féminin (p<10-3). L'âge était également très lié au statut PDV, avec une augmentation des PDV avec l'âge (p<10-3).

Les statuts professionnel et matrimonial étaient respectivement très liés au statut PDV avec un taux maximum de PDV chez les sujets inactifs (64%) et mariés (50%) vs 55% chez les sujets actifs et 34% chez les célibataires (p<10-3). Il existait aussi une association significative du statut PDV avec le niveau socio-économique des patients avec un taux des PDV de 58% chez les patients de bas niveau contre 39% chez les sujets ayant un moyen ou haut niveau socio-économique (p<10-3).

Sur le plan médical, l'existence d'antécédents familiaux de cancer était associée à un taux moindre de PDV (34% vs 49%, p<10-3) Le stade de la maladie était aussi très associé au statut PDV, en effet, au stade 0 ou1 de la maladie le taux des PDV était de 35% puis ce taux augmente progressivement avec respectivement 39%, 56% et 67% pour les stades 2, 3 et 4 (p<10-3).

Le type de traitement reçu était également lié au statut des PDV, ce taux était le plus faible chez les patients ayant reçu un traitement combiné à base de chirurgie, chimiothérapie et radiothérapie (17%) alors que ce taux était à 73% chez les sujets ayant reçu une radiothérapie seule et 52% chez les patients avec chimiothérapie seule, ou encore 57% avec chirurgie seule. (p<10-3).

Un certain nombre de variables n’étaient pas associées au statut PDV: l'origine rurale ou urbaine, le niveau d’éducation et la couverture sociale (respectivement p<0,30, p<0,80 et p<0,12). La présence d'antécédents personnels de cancer n'était pas significativement liée au taux des PDV. (p<0,80). La distance entre la zone de résidence des patients et les lieux de diagnostic et de traitement n'était pas non plus associée au statut PDV.

### Discussion

Nous avons étudié la fréquence des patients pris en charge pour une maladie cancéreuse et perdus de vue après la première année de suivi ainsi que les facteurs liés à ce statut «PDV». Cette étude rétrospective a été réalisée sur un échantillon représentatif de dossiers de patients suivis dans trois centres hospitaliers spécialisés du Maroc.

Notre étude a montré chez ces patients une proportion élevée de PDV. En effet, 48% de la population de notre étude étaient PDV à 1 an de suivi. En Inde, une étude multicentrique menée en 2002 [5] a montré un taux de PDV entre 1 et 3 ans de suivi allant de 0,7% pour le cancer du poumon à 3,2% pour les lymphomes. Dans notre contexte, un taux aussi important des PDV doit nous inciter à repenser la manière du suivi des malades et à s'orienter vers des méthodes actives de suivi des patients atteints de cancer [6]. En effet l'existence de registres de mortalité permet de connaître le statut vital des PDV qui échappent au suivi actif surtout qu'actuellement on pense que la perte de suivi durant la première année de diagnostic est essentiellement due au décès des malades alors que les PDV tardifs sont plutôt des survivants [5].

La proportion des PDV était plus importante chez les patients de sexe masculin. Ce résultat peut être expliqué par la fréquence élevée des cas de cancer du poumon chez les hommes. En effet, 56% des sujets de sexe masculin avaient un cancer du poumon contre 3% chez les femmes (p<10-3) et selon Globocan 2008 le taux de mortalité standardisée sur l’âge du cancer du poumon chez l’homme est de 29,4/100000 Hommes/an contre un taux de 11/100000/an chez la femme [7].

La localisation cancéreuse la plus liée au statut PDV était le poumon suivie par le colon rectum, ce résultat peut être expliqué par une mortalité élevée de ces cancers qui est due à leurs stades tardifs de diagnostic. En effet, 52% des cas de cancer pulmonaire ont été diagnostiqués au stade 4 contre seulement 4% des cas au stade 1 et 2. Pour le cancer colorectal la découverte était faite au stade 4 chez 28% des cas contre 18,5% et 7,2% des cas pour le sein et le col respectivement (p<0.000).

Comme on l'a vu le taux des PDV était significativement associé au stade de diagnostic, en effet, plus ce stade était tardif plus la proportion de PDV était élevée. Il est actuellement reconnu que le stade de diagnostic est un facteur pronostic très important [8, 9] et que la survie des patients en est très liée. D'autre part, le type de traitement reçu était également très associé au statut PDV, or, le traitement dépend directement du stade de diagnostic et de la localisation cancéreuse.

L’âge des patients était également proportionnel aux taux des PDV. Ce résultat concorde avec une large étude réalisée en France en 2006 par le réseau FRANCIM [10] qui a démontré que l’âge était un facteur important de pronostic chez les patients atteints de cancer et qu'il joue en défaveur de ces patients essentiellement pendant la période de suivi précoce comme le cas dans notre étude. Ici encore une corrélation entre les taux des PDV et la mortalité est très probable.

Notre étude a montré aussi que la fréquence des PDV chez les patients à bas niveau socioéconomique était plus élevée que chez les patients à moyen ou à haut niveau socioéconomique. Ce résultat paraît logique en raison du coût très important de la prise en charge et du suivi de la maladie cancéreuse et de l'insuffisance en matière de couverture sociale qui ne couvre actuellement que 17% des dépenses globales de la santé au Maroc [11] alors que seulement 34% des marocains disposent d'une couverture sociale. Une étude réalisée aux Philippines en 2009 [12], dans le but de connaitre les raisons d’être PDV chez des femmes survivantes après un suivi en onco-gynécologie, a montré que les femmes survivantes PDV étaient d'un niveau socio-économique plus bas que celles qui avaient complété leur suivi.

Le statut matrimonial était également lié au statut PDV avec des pertes importantes concernant les sujets mariés et divorcés par rapport aux célibataires. Ceci s'explique par la différence d’âge entre ces catégories du statut matrimonial (les célibataires étant plus jeunes que les sujets mariés) dans la mesure où le taux des PDV augmente avec l’âge.

Un autre résultat important concerne les sujets qui ont eu des antécédents familiaux de cancer, ces derniers étaient moins PDV que les sujets qui n'en avaient pas. Ce résultat peut être lié à la sensibilisation des patients ayants des antécédents familiaux de cancer à la gravité de la maladie et à l'importance de la prise en charge et du suivi.

Contrairement à ce qui était attendu, le statut de PDV n’était pas lié à la distance entre le lieu de résidence et le lieu de TTT des patients. Cependant, il faut considérer ce résultat avec prudence. En effet l'information utilisée a été recueillie sur les dossiers et non par l'interrogatoire des patients. On ne peut exclure que certains patients, par crainte de ne pas être pris en charge dans un des centres de référence étudiés et d’être réorientés vers le centre hospitalier de leur région d'origine, aient déclaré comme lieu de résidence les adresses de leurs hôtes éventuels qui se situaient dans la même ville que les centres de prise en charge.

Notre étude présente cependant un certain nombre de limites. Nous avons mené notre étude dans trois centres du secteur publique; en effet, jusqu′en 2007 ces trois centres représentent les seuls centres publics de soins pour le cancer au Maroc. En outre, le secteur privé (représenté, jusqu′en 2007, par trois cliniques privées: deux à Casablanca et une à Rabat) recrute une très faible proportion des cas de cancer au Maroc. Comme nous avons travaillé sur les dossiers des hôpitaux, la période entre 2003 et 2007, nous croyons que notre population peut être considéré comme représentative des cas de cancer qui ont accès au système de santé au Maroc. Les données ont été recueillies à partir de dossiers d'hospitalisation et non directement auprès des patients eux-mêmes. De ce fait nous nous sommes trouvés confrontés à un nombre élevé de données manquantes pour les variables étudiées, notamment la profession, le niveau d’étude et la couverture sociale. Cette situation ne nous a pas permis de réaliser, comme nous l'aurions souhaité, une analyse multivariée qui aurait permis d’éviter les croisements entre variables. Surtout, enfin, l'absence de registres de mortalité n'a pas permis de connaître le statut vital des patients perdus de vue.

L'intérêt de ces premiers résultats nous a conduits à développer le protocole d'une nouvelle étude visant à recueillir directement l'information sur le devenir des patients PDV dans l’étude initiale. En utilisant les coordonnées des patients PDV, il sera alors possible de récupérer activement les informations relatives à leur devenir et à rechercher les causes de leur statut de PDV.

D'ores et déjà les résultats du présent travail, incitent à réfléchir à la mise en place rapide de mesures de nature à favoriser un suivi actif des patients atteints de cancer. Ceci passe par la généralisation des registres de cancer et de mortalité sans lesquels il serait impossible de mesurer avec précision le vrai poids du problème de suivi. Enfin cette étude montre la nécessité d'une sensibilisation large et systématique de la population au problème du cancer, mais aussi de l'extension de la couverture sociale, de l’élargissement du réseau de soins à tout le territoire et du dépistage précoce des cancers les plus fréquents au Maroc.

## Conclusion

Notre étude constitue le premier travail réalisé au Maroc sur ce problème des PDV chez les patients atteints de cancer. Ces résultats ont mis en évidence la grande ampleur de ce phénomène. Cette situation préjudiciable aux patients devrait inciter tous les professionnels de santé æuvrants dans le domaine de la cancérologie (cliniciens médecins de santé publique, décideurs) à collaborer ensemble pour prendre les mesures qui s'imposent pour y pallier.
